# *Beta-2-Microglobulin* Regulates Sheep Susceptibility to *Escherichia coli* *F17b* in Intestinal Epithelial Cells

**DOI:** 10.3390/vetsci13030252

**Published:** 2026-03-09

**Authors:** Xinyu Gu, Weihao Chen, Hadeer M. Aboshady, Ahmed A. Saleh, Yuxuan Song, Xiyun Zhang, Hossam E. Rushdi, Wei Sun

**Affiliations:** 1 College of Animal Science and Technology, Yangzhou University, Yangzhou 225009, China; g2940994463@163.com (X.G.); 18552133709@163.com (W.C.); 2 Department of Animal Production, Faculty of Agriculture, Cairo University, Algammaa Street, Giza 12613, Egypt; hadeer.morsy@agr.cu.edu.eg; 3 Animal and Fish Production Department, Faculty of Agriculture (Al-Shatby), Alexandria University, Alexandria City 11865, Egypt; ahmedabdulqadersaleh@alexu.edu.eg; 4 College of Animal Science and Technology, Northwest A&F University, Yangling, Xianyang 712100, China; syx98728@163.com; 5 Gansu Yuansheng Agriculture & Animal Husbandry Technology Co., Ltd., Jinchang 737200, China; ycys_zxy@163.com; 6 International Joint Reserarch Laboratory in Universities of Jiangsu Province of China for Domestic Animal Germplasm Resources and Gentic Improvement, Yangzhou University, Yangzhou 225009, China; 7 Joint International Research Laboratory of Agriculture and Agri-Product Safety of Ministry of Education of China, Yangzhou University, Yangzhou 225009, China; 8 “Belt and Road Initiative” Academy of Evaluation, Protection, and Improvement on Sheep Resources, Ministry of Agriculture and Rural Affairs of China, Yangzhou 225009, China

**Keywords:** sheep, *Beta-2-microglobulin*, *E. coli F17b*, intestinal epithelial cells

## Abstract

*Beta-2-microglobulin* (B2M) is a key component protein in the processing and presentation of major histocompatibility complex (MHC)-I antigens and plays an important role in the immune system regulation. Previous studies have shown that B2M is significantly overexpressed in the intestinal tissues of sheep that are resistant to *E. coli F17b* infection (defined by milder clinical symptoms post-challenge) compared to those that are susceptible (exhibiting severe diarrhea). Based on this finding, this study aimed to investigate whether B2M influences the adhesion of *E. coli F17b* to sheep intestinal epithelial cells (IECs) and to assess its role in regulating IEC proliferation and migration. We tested this by overexpressing and knocking down B2M in IECs, and then measured bacterial adhesion through colony counts and fimbrial gene expression (RT-qPCR). Moreover, cell health was assessed using proliferation (CCK-8 and EdU) and migration (scratch) assays. The results showed that upregulation of B2M expression inhibited *E. coli F17b* adhesion and promoted IEC proliferation and migration. Silencing B2M increased bacterial adhesion and impaired cell function. In summary, B2M helps protect sheep IECs from *E. coli F17b* by strengthening the epithelial barrier through improved cell growth, proliferation, and migration. These findings elucidate part of the host defense mechanism against *E. coli F17b*, providing a basis for further research.

## Simple Summary

This study investigated how Beta-2-microglobulin affects sheep susceptibility to Escherichia coli F17b infections in intestinal epithelial cells. The main goal was to determine whether Beta-2-microglobulin influences bacterial adhesion and the function of intestinal epithelial cells. Results showed that Beta-2-microglobulin overexpression reduced Escherichia coli F17b adhesion and promoted intestinal epithelial cells proliferation and migration, while Beta-2-microglobulin knockdown increased adhesion and impaired cell function. The study concludes that Beta-2-microglobulin protects sheep intestinal epithelial cells by strengthening the epithelial barrier, offering insights for a potential breeding disease-resistant sheep and improving livestock health, thereby reducing economic losses in animal farming.

## 1. Introduction

*Escherichia coli expressing F17* fimbriae is an important pathogen associated with diarrhea in calves and lambs, leading to significant economic losses [1]. F17 fimbriae mediate bacterial adhesion to host small intestinal epithelial cells (IECs), facilitating colonization, which is a critical prerequisite for the delivery of virulence factors [2]. The F17 family comprises several variants, including F17a, F17b, F17c, and F17d, which are found across different pathogenic *E. coli* pathotypes [3,4].

The classical pathogenesis of ETEC involves a two-step mechanism: initial adhesion via fimbriae to the intestinal epithelium, followed by the secretion of heat-labile (LT) and/or heat-stable (ST) enterotoxins, which disrupt ion and water homeostasis in IECs, leading to secretory diarrhea [5]. Animal ETEC isolates commonly produce STa, STb, and/or EAST1 enterotoxins, with F17 fimbriae serving as important colonization factors in calves and lambs [1,6]. In our previous studies, we identified lambs with divergent susceptibility to *E. coli F17b* infection through challenge experiments: susceptible lambs developed typical diarrheic pathological changes such as yellow watery feces, intestinal congestion, and liver enlargement, while resistant lambs exhibited milder lesions [7].

*Beta-2-microglobulin* (B2M) is a 12 kDa protein component of MHC class I molecules, involved in antigen presentation and immune regulation [8]. Beyond its immunological roles, B2M exhibits antimicrobial properties and has been implicated in innate defense against bacterial infections [9]. A previous transcriptomic study reported higher B2M expression in the intestines of sheep resistant to *E. coli F17b* compared to susceptible individuals [10], suggesting a potential protective role. In our previous studies, susceptible lambs and antagonistic lambs were identified, the specific method was to use feeding to carry out the *E. coli F17* challenge test, the high-dose challenge group, the low-dose challenge group, and the positive control group were given 10 mL of *E. coli* bacterial solution by gavage every day, and the negative control group was fed the same amount of normal saline for 4 days (4 days of age–7 days of age), and the susceptible lambs showed typical diarrhea pathological changes: such as yellow watery feces, anal fecal residue, liver enlargement, intestinal congestion and other symptoms, The degree of lesion in antagonistic lambs is relatively weak [7]. As an essential component of MHC-I, B2M is also highly expressed in antigen-presenting cells (APCs), such as macrophages and dendritic cells, where it is crucial for presenting pathogen-derived peptides to CD8+ T cells to initiate adaptive immunity. Recent studies suggest B2M may have broader immunomodulatory roles beyond antigen presentation, potentially influencing APC function itself [11,12]. However, the direct function of B2M in modulating *E. coli F17b* infection in sheep IECs remains unexplored.

We hypothesize that B2M enhances the resistance of sheep IECs to *E. coli F17b* infection by inhibiting bacterial adhesion and promoting epithelial repair through enhanced cell proliferation and migration. Therefore, this study aimed to investigate whether B2M influences the adhesion of *E. coli F17* (F17b subtype) to sheep IECs and to assess its role in regulating IEC proliferation and migration, thereby elucidating potential mechanisms of host resistance.

## 2. Materials and Methods

### 2.1. Bacterial Strain, Experimental Animals and Cell Culture

The *Escherichia coli* strain used was DN1401, which expresses F17b fimbriae and was originally isolated from a diarrheic calf (obtained from the School of Animal Medicine, Northeast Agricultural University). Whole-genome sequencing of this strain was performed in our laboratory (unpublished data), and analysis confirmed the presence of the astA, paa, and papC genes, while all classical ETEC enterotoxin genes (eltA, eltB, estA, estB) and all NTEC-associated toxin genes (cnf1, cnf2, cnf3, cdt-III, cdt-IV, afa operon) were absent (Table S1). Bacterial cultures were grown in LB medium, and colony-forming units (CFU) were quantified by plate counting for infection assays.

Primary intestinal epithelial cells (IECs) were isolated from the jejunum of 3–5-day-old healthy Hu sheep (Jiangsu Xilaiyuan Ecological Agriculture Co., Ltd., Taizhou, China) as described previously [13]. The cells were characterized by positive immunofluorescence staining for cytokeratin 18 (CK18) and were used at passages 3–6 to maintain their primary phenotype. Cells were cultured in DMEM/F12 medium (HyClone, Logan, UT, USA) supplemented with 10% fetal bovine serum and 1% penicillin–streptomycin at 37 °C with 5% CO_2_.

### 2.2. Experimental Design and Replication

A fully defined in vitro experimental design was employed. The study comprised four main treatment groups for functional assays: (1) Negative Control: Untreated IECs or cells transfected with empty vector (pcDNA3.1(+)) or non-targeting siRNA (siRNA-NC); (2) *E. coli F17b* Infection Group; (3) B2M Overexpression Group; (4) B2M Knockdown Group. For bacterial adhesion assays, a multiplicity of infection (MOI) of 100:1 (bacteria:cells) was used. This MOI was determined through preliminary optimization experiments to ensure measurable adhesion while minimizing excessive cytotoxicity. The number of adherent bacteria may appear limited in an in vitro setting due to the absence of complex mucosal and immune factors present in vivo, the relatively short infection period (3 h), and the use of a standardized, non-saturating bacterial concentration to specifically assess the adhesive interaction. All key assays were performed with independent biological replicates: EdU assay (n = 17 independent wells), bacterial adhesion assay (n = 3 independent experiments, each with duplicate technical replicates), CCK-8 assay (n = 3 independent experiments), and scratch wound healing assay (n = 3 independent experiments). Detailed sample sizes are also indicated in the corresponding figure legends.

### 2.3. RNA Extraction and Quantitative Real-Time PCR (RT-qPCR)

Total RNA was extracted using *TRIzol* reagent (Tiangen, Beijing, China) following the manufacturer’s protocol. Briefly, cells in a 6-well plate were lysed directly with 1 mL TRIzol per well. The homogenate was transferred to a nuclease-free microcentrifuge tube, mixed with 0.2 mL chloroform, shaken vigorously for 15 s, and incubated at room temperature for 3 min. After centrifugation at 12,000× *g* for 15 min at 4 °C, the aqueous phase was transferred to a new tube. RNA was precipitated with 0.5 mL isopropanol, washed with 75% ethanol, air-dried, and dissolved in RNase-free water. RNA quality was assessed spectrophotometrically (A260/A280 ratio 1.8–2.0). cDNA was synthesized using a reverse transcription kit. RT-qPCR was performed with gene-specific primers (Table 1) using SYBR Green Master Mix on a QuantStudio system. The 2*^−^*^ΔΔCt^ method was used for relative quantification [14], with GAPDH as the endogenous control. The online tool Primer-BLAST (NCBI, https://www.ncbi.nlm.nih.gov/tools/primer-blast/, accessed on 15 October 2023) was used for primer design.

### 2.4. Bacterial Adhesion Assay

IECs were seeded in 24-well plates at a density of 5 × 10^4^ cells per well and transfected as described above. After 24 h, the growth medium was aspirated and the cells were gently washed once with sterile PBS. Fresh, antibiotic- and serum-free F12 medium was added. The *E. coli* *F17b* bacterial suspension was centrifuged, washed with PBS, and resuspended in F12 medium. The bacterial suspension was then added to each well to achieve a predetermined MOI of 100:1. The plates were centrifuged at 600× *g* for 5 min to synchronize the bacteria in contact with the cells, followed by incubation at 37 °C at 5% CO_2_ for 3 h.

After incubation, the supernatant containing non-adherent bacteria was carefully aspirated. To completely remove loosely attached bacteria, the cell monolayer was gently washed three times with 1 mL of sterile PBS for 5 min per wash. To lyse IECs and release adherent bacteria, 200 μL of PBS containing 0.5% Triton X-100 was added to each well, and the plates were incubated at 37 °C for 30 min with occasional tapping to ensure adequate lysis. The lysate was transferred to a 2 mL sterile centrifuge tube. The wells were rinsed three times with 1 mL of PBS, and the rinsates were combined with the corresponding lysate, resulting in a total volume of 3.2 mL.

The collected suspension was serially diluted 10-fold in PBS. In preliminary experiments, dilutions of 10^−4^, 10^−5^, and 10^−6^ were tested. The 10^−4^ dilution produced too many colonies to count accurately, while the 10^−6^ dilution yielded too few colonies. The 10^−5^ dilution consistently gave 20–200 colonies per plate and was therefore selected for all subsequent adhesion assays. From this dilution, 500 μL was spread onto 3M Petrifilm Aerobic Count Plates (3M, St. Paul, MN, USA) in duplicate. The Petrifilms were incubated at 37 °C for 18 h, and colonies were counted using ImageJ software (v1.53). The colony-forming units (CFU) per well were calculated from these raw colony counts using the following formula for converting the data:

$$CFU/well=colony\;count\times 10^{5}\times \frac{3.2}{0.5}$$

For statistical analysis, CFU/well values were log_10_-transformed. Data are presented as mean ± SD of log_10_ CFU per well from three independent biological replicates.

At the same time, a part of the lysate was taken and bacterial genomic DNA was extracted using a commercial kit, and the expression levels of F17b fimbrial genes (F17, F17b-A, F17b-G) were quantified by RT-qPCR.

### 2.5. Cell Proliferation Assay

Cell proliferation was assessed using CCK-8 and EdU assays. For CCK-8, transfected IECs were seeded in 96-well plates. At 0, 24, 48, and 72 h post-transfection, 10 µL of CCK-8 reagent (Tecan, Shanghai, China) was added per well, incubated for 2 h, and absorbance at 450 nm was measured using a microplate reader (Tecan, Männedorf, Switzerland). For EdU assay, cells grown on coverslips in 24-well plates were treated with 10 µM EdU (RiboBio, Guangzhou, China) for 2 h, fixed, and stained using the Cell-Light EdU Apollo 567 kit according to the manufacturer’s instructions. EdU-positive cells were visualized and counted under an inverted fluorescence microscope (Nikon Eclipse Ti2, Tokyo, Japan) using a 20× objective lens.

### 2.6. Cell Migration Assay

A scratch wound healing assay was performed. Transfected IECs were grown to confluence in 6-well plates. A sterile 200 µL pipette tip was used to create a uniform scratch. The scratch was immediately washed twice with PBS to remove dislodged cells. Cells were then incubated in serum-free medium to minimize proliferation-based closure. Scratch images were captured at 0, 12, and 24 h using a phase-contrast microscope (Nikon Eclipse Ts2, Tokyo, Japan) with a 4× objective lens. Migration area and scratch width were quantified using ImageJ software (v1.53).

### 2.7. Statistical Analysis

Data were analyzed using SPSS 26.0 (IBM Corp., Armonk, NY, USA), and graphs were generated with GraphPad Prism 9.5. All quantitative data are presented as mean ± standard deviation (SD). Normality of data was tested using the Kolmogorov–Smirnov test. Comparisons between two independent groups were performed using Student’s *t*-test. Comparisons among multiple groups were analyzed using one-way analysis of variance (ANOVA) followed by Duncan’s multiple range test for post hoc analysis. Statistical significance is explicitly denoted in all figures: ** *p* < 0.05, * *p* < 0.01. The sample sizes for each assay are as detailed in Section 2.2.

## 3. Results

### 3.1. Construction of BMDPI2M Overexpression Vector and Evaluation of Its Overexpression and Knockdown Efficiency

The B2M overexpression vector was synthesized by Tsingke Company (Beijing, China), based on the B2M sequence downloaded from NCBI. The target vector was pcDNA3.1(+), with cloning sites at 5′ HindIII and 3′ BamHI. Following transformation and plating, plasmids were extracted after 16 h. Successful cloning was confirmed through dual digestion with Quick Cut HindIII (Takara, Osaka, Japan) and Quick Cut BamHI (Takara, Japan). The primer sequences used for B2M were as follows:

pcDNA3.1(+)-B2M-F:ctagcgtttaaacttaagcttATGGCTGTCTCCGCGGCC

pcDNA3.1(+)-B2M-R:aacgggccctctagactcgagTTAGAGGTCTCGATCCCACTTAACT

The lowercase letters represent the added restriction enzyme sites and protective bases.

On the other hand, siRNA-B2M was designed and synthesized by Shanghai Jima Pharmaceutical Co., Ltd. (Shanghai, China), with the sequences listed in Table 2.

The overexpression vector of B2M was synthesized by Tsingke Company (Beijing, China). Transformed bacteria were inoculated into Luria–Bertani (LB) medium containing A+, and after overnight incubation for 12 h, single colonies were selected and transferred to 1 mL of LB medium. After 6 h of shaking at 37 °C, plasmids were extracted. Successful cloning was confirmed by dual digestion with BamHI and HindIII, resulting in distinct bands at 5082 bp and 346 bp (Figure S1), indicating the successful construction of the pcDNA3.1(+)-B2M overexpression vector.

IECs were transfected with pcDNA3.1(+)-B2M, and RT-qPCR was conducted to assess the mRNA expression levels of B2M. The results showed a significant (*p* < 0.01) increase in the mRNA expression of B2M (Figure S2), confirming the suitability of this plasmid for subsequent experiments.

Three different Small Interfering RNAs (siRNAs) of B2M were used to transfect IECs, and RT-qPCR was performed to measure B2M mRNA expression levels. The siRNA-322 group exhibited the highest significant (*p* < 0.01) reduction in B2M mRNA expression (Figure S3). Based on these results, siRNA-322 was selected for subsequent analyses.

### 3.2. Overexpression of B2M Reduces E. coli F17b Adhesion to IECs

**Figure 1:**
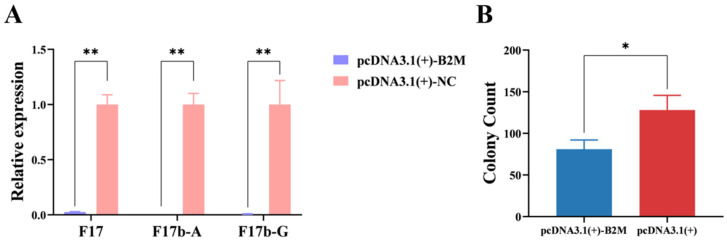
Effects of B2M overexpression on *E. coli F17b* adhesion. (**A**) mRNA expression levels of fimbrial genes following B2M overexpression. (**B**) Adhesion of *E. coli F17b* to IECs after B2M overexpression, measured as colony count (per 500 μL of 10^−5^ dilution). Values are mean ± SD (n = 3). * *p* < 0.05, ** *p* < 0.01.

To analyze the impact of B2M on the adhesion of *E. coli F17b* to IECs, F17b was allowed to adhere to IECs for 3 h, after which RT-qPCR was conducted to measure the expression levels of F17b fimbrial genes post B2M overexpression and interference. B2M overexpression significantly (*p* < 0.01) reduced the mRNA levels of F17, F17b-A, and F17b-G fimbriae (Figure 1A). Additionally, the log_10_ CFU per well of *E. coli F17b* was significantly (*p* < 0.05) lower in the B2M overexpression group compared to the control group (Figure 1B).

**Figure 2:**
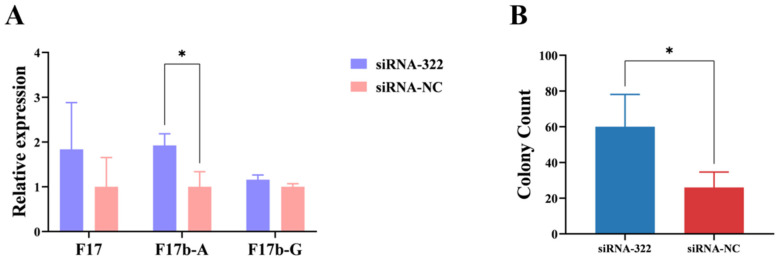
Effects of B2M interference on *E. coli F17b* adhesion. (**A**) mRNA expression levels of fimbrial genes following B2M interference. (**B**) Adhesion of *E. coli F17b* to IECs after B2M interference, measured as colony count (per 500 μL of 10^−5^ dilution). Values are mean ± SD (n = 3). * *p* < 0.05.

Following B2M interference, the mRNA expression levels of F17b-A fimbriae were increased significantly (*p* < 0.05) (Figure 2A). Moreover, the log_10_ CFU per well were significantly (*p* < 0.05) higher compared to the control group (Figure 2B). These results reveal that B2M gene can inhibit the adhesion of *E. coli F17b* to IECs.

### 3.3. B2M Promotes Proliferation of IECs

**Figure 3:**
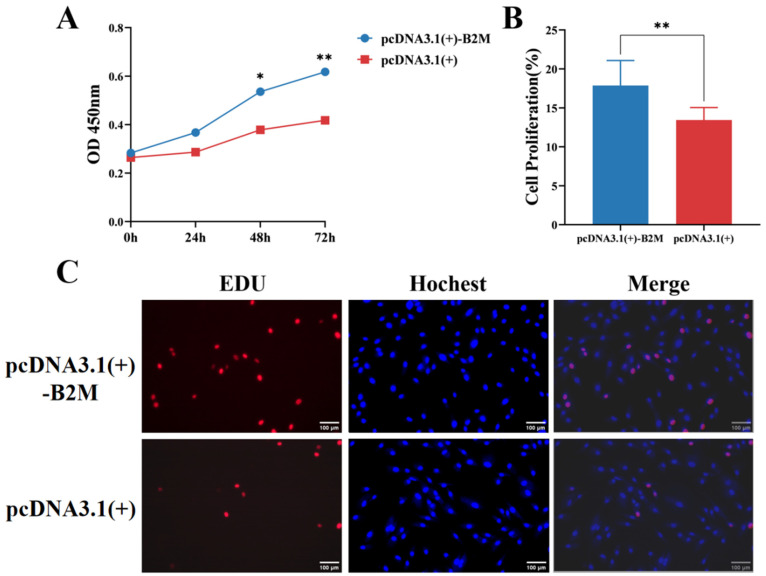
B2M overexpression promotes proliferation of IECs. (**A**) OD450 values from CCK-8 analysis after B2M overexpression. Data are presented as the mean ± SD of three independent biological replicates (n = 3). (**B**) Proportion of EdU-positive Hu sheep IECs. Data are presented as the mean ± SD of seventeen independent replicates (n = 17). (**C**) Number of proliferating IECs detected by EdU assay after B2M overexpression. EdU staining (red) indicates cells in the proliferation phase; Hoechst staining (blue) shows the cell nuclei. The image scale is 200×. * *p* < 0.05, ** *p* < 0.01. Data are presented as the mean ± SD of seventeen independent replicates (n = 17).

The potential impact of B2M overexpression on the proliferation of IECs was estimated. The CCK-8 assay was employed to assess the proliferation of IECs. After transfecting IECs with the B2M overexpression plasmid for 48 h, the proliferation rate was significantly higher than that of the control group (*p* < 0.05), increasing gradually to reach its peak at 72 h (*p* < 0.01) (Figure 3A). Furthermore, following B2M overexpression, the number of EdU-positive cells significantly increased (*p* < 0.01) in the treated group compared to the control one (Figure 3B). These findings indicate that B2M overexpression promotes the proliferation of IECs.

**Figure 4:**
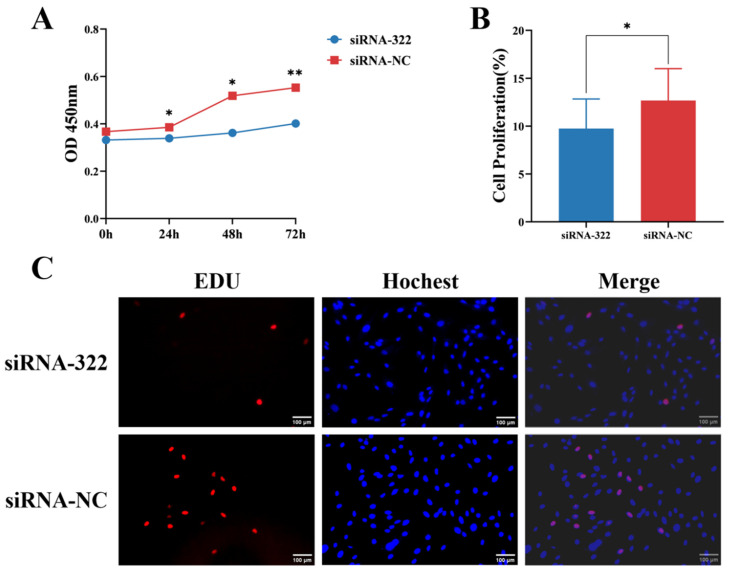
B2M interference inhibits proliferation of IECs. (**A**) OD450 values from CCK-8 analysis following B2M interference. Data are presented as the mean ± SD of three independent biological replicates (n = 3). (**B**) Proportion of EdU-positive Hu Sheep IECs. Data are presented as the mean ± SD of seventeen independent replicates (n = 17). (**C**) Number of proliferating IECs detected by EdU assay after B2M interference. EdU staining (red) indicates cells in the proliferation phase; Hoechst staining (blue) shows the cell nuclei. The image scale is 200×. * *p* < 0.05, ** *p* < 0.01. Data are presented as the mean ± SD of seventeen independent replicates (n = 17).

Additionally, IECs were transfected with B2M interference siRNA. After B2M interference, CCK-8 assays revealed that the proliferation rates at 24 and 48 h post-transfection were significantly (*p* < 0.05) lower than those of the control group. This difference became highly significant (*p* < 0.01) at 72 h post-transfection (Figure 4A). Furthermore, the number of EdU-positive cells significantly (*p* < 0.05) decreased following B2M interference (Figure 4B). These results indicate that B2M interference can inhibit the proliferation of IECs.

### 3.4. B2M Promotes Migration of IECs

**Figure 5:**
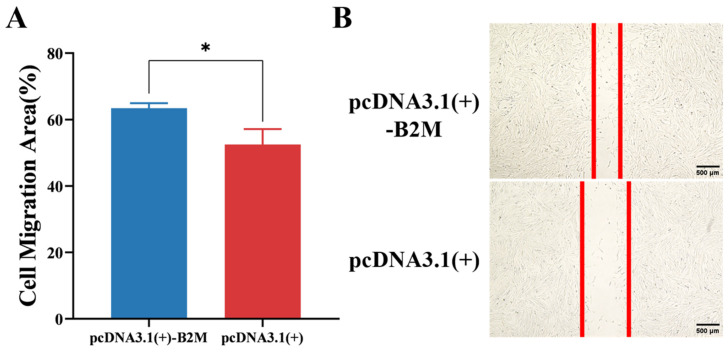
B2M Overexpression Promotes Migration of IECs. (**A**) Migration area of IECs following B2M overexpression. (**B**) Width of the scratch after B2M overexpression. The image scale is 40×. * *p* < 0.05. Data are presented as the mean ± SD of three independent biological replicates (n = 3).

The effect of B2M overexpression on the migration of IECs was investigated using a scratch assay. After transfecting IECs with the B2M overexpression plasmid for 60 h, scratches were made, and the cells were incubated at 37 °C for 24 h. The migration area of IECs was significantly (*p* < 0.05) larger than that of the control group (Figure 5A). On the other hand, the width of the scratch was significantly (*p* < 0.05) smaller than that of the control group (Figure 5B). These findings imply that B2M overexpression promotes the migration of IECs.

**Figure 6:**
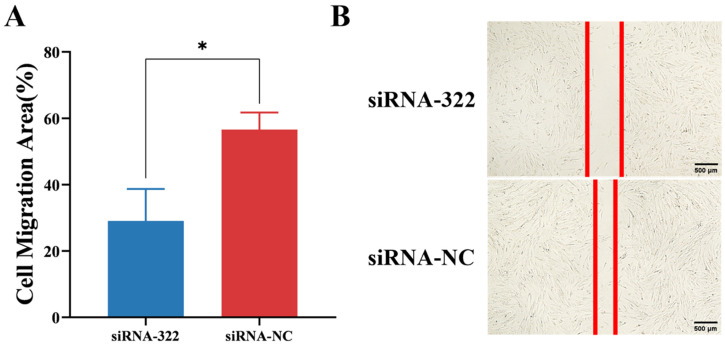
B2M interference inhibits migration of IECs. (**A**) Migration area of IECs following B2M interference. (**B**) Width of the scratch after B2M interference. The image scale is 40×. * *p* < 0.05. Data are presented as the mean ± SD of three independent biological replicates (n = 3).

Furthermore, IECs were transfected with B2M interference siRNA. After 60 h of transfection and 24 h of incubation post-scratch, the migration area of the cells was significantly (*p* < 0.05) lower than that of the control group (Figure 6A). However, the width of the scratch was significantly greater than that of the control group (*p* < 0.05) (Figure 6B). Obviously, these results indicate that B2M interference can inhibit the migration of IECs.

## 4. Discussion

This study demonstrates that B2M plays a protective role in sheep IECs against *E. coli F17b* infection through a dual mechanism: inhibiting bacterial adhesion and promoting epithelial repair via enhanced proliferation and migration. The primary aim of this work was to determine whether B2M influences F17b adhesion and IEC repair functions. Our results align with this hypothesis, confirming B2M’s regulatory role in these processes.

*E. coli* is a significant pathogen associated with acute diarrhea in farm animals [15]. Diarrhea has a crucial impact on the survival rate of lambs, especially in newly born and suckling lambs. Diarrhea not only directly causes lamb mortality, but may also indirectly affects survival rates by triggering secondary diseases, malnutrition, and growth retardation. Therefore, it is crucial to study the pathogenesis of *E. coli F17b* and the host defense mechanisms to enable effective infection control. *E. coli F17b* can adhere to intestinal epithelium via fimbriae, recognizing specific receptors on the small intestine, thereby colonizing this habitat [2].

F17 fimbriae are produced by diverse pathogenic *E. coli* strains in domestic animals. As reviewed by Le Bouguénec and Bertin, the F17 family encompasses variants such as F17a (often linked to EPEC/ETEC in calves) and F17b (frequently isolated from NTEC strains producing CNF2) [16]. The strain used in this study, DN1401, carries the F17b fimbrial operon together with the astA, paa, and papC genes. It lacks all classical ETEC enterotoxin genes (eltA, eltB, estA, estB) and all NTEC-associated toxin genes (cnf1, cnf2, cnf3, cdt-III, cdt-IV, afa). Among the detected genes, astA (encoding EAST1) and paa (porcine attaching–effacing-associated protein) are recognized as accessory virulence factors in ETEC, contributing to enterotoxicity and adhesion, respectively [1,6,17]. The papC gene, typically associated with P fimbriae in uropathogenic *E. coli*, is not a common ETEC marker but may contribute to adhesion in certain contexts [18]. Given that the strain possesses F17b fimbriae (which mediate binding to intestinal receptors [2]) together with ETEC-associated accessory factors astA and paa, but lacks the hallmark ETEC enterotoxin genes (LT/ST) and all NTEC toxins, we propose that DN1401 may represent a non-classical ETEC strain. Its ability to cause diarrhea in lambs [7] could be attributed to the combined action of these accessory factors and its strong adhesive properties. Further studies are warranted to elucidate the precise role of paa and papC in the pathogenesis of this strain and to explore whether additional, yet-unidentified virulence genes contribute to its phenotype.

B2M is an important subunit of the MHC class I, a non-glycosylated protein of 11.6 kDa found on the surface of all nucleated cells [19]. MHC class I molecules play a critical role in alerting the immune system to the presence of virus-infected cells [20]. Our findings demonstrate that upregulation of B2M expression can suppress the adhesion of *E. coli F17b* to IECs, consistent with previous studies [21,22]. Moreover, B2M displays antibacterial activity in animals and humans. B2M found in human amniotic fluid shows broad antibacterial activity against *Listeria monocytogenes*, *E. coli*, and *Staphylococcus aureus* [21]. During bacterial infections in mice, B2M-dependent mechanisms are involved in innate defense [22]. Thus, we speculate that the antibacterial capability of B2M is widespread across species. The correlation we observed between host B2M levels and bacterial fimbrial gene expression suggests that B2M may alter the host cell surface or microenvironment in a way that disfavors the expression or function of F17b adhesins, a potential novel aspect of host–pathogen interaction at the molecular level.

Further investigations revealed that B2M exerts a bidirectional regulatory effect on the biological functions of IECs, including both proliferation- and migration-promoting effects. Results from CCK-8 and EdU assays demonstrated that B2M overexpression significantly enhanced cell proliferation, whereas B2M knockdown markedly suppressed it. These findings align with reports that B2M promotes the growth of human prostate cancer cells, suggesting B2M may enhance proliferative capacity by activating cell cycle-related pathways, such as PI3K/Akt [23]. Regarding migration, scratch assays demonstrated that cell migration was significantly enhanced in the B2M overexpression group, while impaired in the knockdown group. This observation aligns with findings that B2M promotes wound healing via fibroblast activation during cardiac injury repair, indicating B2M may enhance cell migration through regulating cytoskeletal proteins or matrix metalloproteinases [24].

To the best of our knowledge, this is the first study to demonstrate in sheep IECs that B2M counteracts F17b infection through a dual mechanism of inhibiting bacterial adhesion and promoting epithelial repair. Furthermore, as an essential component of MHC-I, B2M is ubiquitously expressed, including in immune cells like macrophages and dendritic cells, where it is crucial for antigen presentation and the activation of cytotoxic T lymphocytes [8]. Emerging research suggests B2M’s role in immunity may be multifaceted. For instance, high concentrations of B2M have been reported to inhibit the in vitro generation of functional dendritic cells, potentially acting as a negative feedback regulator [11]. Conversely, internalization of B2M aggregates by macrophages can activate the NLRP3 inflammasome, leading to pro-inflammatory cytokine release [12]. These findings indicate that B2M may function as a signaling molecule influencing APC activity. While our study focused on its epithelial-centric functions, the potential for B2M to modulate the broader immune response against F17b infection, possibly through affecting APC function and inflammation, presents an intriguing avenue for future in vivo investigation.

Several limitations of the present study should be acknowledged. First, we focused on proliferation and migration as indicators of epithelial repair but did not assess the expression of tight junction proteins, which are critical for barrier integrity. Future work should include this aspect. Second, the functional validation of B2M in this study was conducted entirely at the cellular level, including its effects on bacterial adhesion, cell proliferation, and migration. While these in vitro approaches provide valuable insights into the potential role of individual genes, the cellular microenvironment cannot fully replicate the complexity of an intact living organism. Differences in immune responses, systemic regulation, and tissue-level interactions in vivo perhaps lead to outcomes that differ from our cellular observations. Therefore, future studies should extend these findings to animal models—such as challenged Hu sheep—to comprehensively evaluate the role of B2M in host resistance against *E. coli F17b* under physiological conditions and to assess its potential as a target for breeding or therapeutic strategies. In this context, our group has previously established a challenge model that successfully identified lambs with divergent susceptibility to *E. coli F17b* [7], and recent integrated multi-omics analyses further revealed key metabolic and transcriptional markers associated with F17b infection in sheep [25]. These studies underscore the importance of combining in vitro mechanistic insights with in vivo validation to fully understand host–pathogen interactions and to identify reliable biomarkers for disease resistance.

We have not yet performed in vivo studies to validate these functions in live sheep. Therefore, future work should focus on extending these findings to animal models to comprehensively evaluate the role of B2M in the host resistance against *E. coli F17b* under normal conditions. In particular, the integration of transcriptomic, metabolomic, and microbiomic data from resistant and susceptible animals could provide a systems-level understanding of how B2M modulates the intestinal microenvironment and host defense networks [25]. Such integrative approaches may also facilitate the development of novel strategies for enhancing disease resistance in livestock, such as through selective breeding or targeted nutritional interventions.

## 5. Conclusions

In conclusion, this study provides evidence that B2M enhances the resistance of sheep intestinal epithelial cells to *E. coli F17b* by reducing bacterial adhesion and promoting epithelial repair through increased proliferation and migration. These findings highlight the multifaceted role of B2M in host defense and repair mechanisms. Future research on living organisms, including sheep breeds, could deal with elucidating the precise molecular pathways through which B2M exerts these effects. Furthermore, evaluating the balance between antibacterial efficacy and possible cytotoxic effects of B2M will be crucial for assessing B2M as a potential therapeutic target or a biomarker for enhancing disease resistance in livestock.

## Figures and Tables

**Table 1:** Sequence information of genes used in RT-qPCR.

**Gene**	**Sequences (5′ → 3′)**	**Product Length (bp)**	**Accession No.**
B2M	F:ACCCGCCAGAAGATGGAAAG	170	XM_060418694
	R:GAACTCAGCGTGGGACAGAA		
F17b-A	F:CAACTAACGGGATGTACAGTTTC	323	L14318.1
	R:CTGATAAGCGATGGTGTAATTAAC		
F17b-G	F:CGTGGGAAATTATCTATCAACG	615	L14319.1
	R:TGTTGATATTCCGTTAACCGTAC		
GAPDH	F: TCTCAAGGGCATTCTAGGCTAC	151	XM_060411593.1
	R: GCCGAATTCATTGTCGTACCAG		
F17	F: GGGCTGACAGAGGAGGTGGGGC	408	
	R: CCCGGCGACAACTTCATCACCGG		

**Table 2:** Primer information.

**Group**	**Forward Primer (5′-3′)**	**Reverse Primer (5′-3′)**
siRNA-105	GAGGUCCAGGUAUACUCAATT	UUGAGUAUACCUGGACCUCTT
siRNA-260	GGACUGGUCUUUCUACCUUTT	AAGGUAGAAAGACCAGUCCTT
siRNA-322	GCUGCCGAGUGAAUCACGUTT	ACGUGAUUCACUCGGCAGCTT
siRNA-NC	UUCUCCGAACGUGUCACGUTT	ACGUGACACGUUCGGAGAATT

## Data Availability

The original contributions presented in this study are included in the article/Supplementary Materials. Further inquiries can be directed to the corresponding authors.

## Supplementary Materials

The following supporting information can be downloaded at: https://www.mdpi.com/article/10.3390/vetsci13030252/s1, Table S1: Virulence gene profile of *E. coli F17b* strain DN1401 based on whole-genome sequencing; Figure S1: Products of the pcDNA3.1(+)-B2M vector after dual digestion with BamHI and HindIII; Figure S2: mRNA expression levels of B2M in Hu sheep IECs following overexpression. * *p* < 0.05, ** *p* < 0.01. Data are presented as the mean ± SD of three independent biological replicates (n = 3); Figure S3: Levels of B2M mRNA expression. * *p* < 0.05, ** *p* < 0.01. Data are presented as the mean ± SD of three independent biological replicates (n = 3).

## Abbreviations

The following abbreviations are used in this manuscript:

B2M	Beta-2-microglobulin
*E. coli F17b*	Escherichia coli F17b
IECs	Intestinal epithelial cells
MHC	major histocompatibility complex
ETEC	enterotoxigenic *E. coli*
CTL	cytotoxic T lymphocyte
AMP	an antimicrobial peptide
CCK-8	Cell Counting Kit-8
EDU	5-ethynyl-2-deoxyuridine

## References

[1] Dubreuil J.D., Isaacson R.E., Schifferli D.M. (2016). Animal Enterotoxigenic *Escherichia coli*. EcoSal Plus.

[2] Kaper J., Nataro J., Mobley H. (2004). Pathogenic *Escherichia coli*. Nat. Rev. Microbiol..

[3] Lintermans P.F., Pohl P., Bertels A., Charlier G., Vandekerckhove J., Vandamme J., Schoup J., Schlicker C., Korhonen T., De Greve H. (1988). Characterization and purification of the F17 adhesin on the surface of bovine enteropathogenic and septicemic *Escherichia coli*. Am. J. Vet. Res..

[4] Bertels A., Pohl P., Schlicker C., Van Driessche E., Charlier G., De Greve H., Lintermans P. (1989). Isolation of the *F*111 fimbrial antigen on the surface of a bovine *Escherichia coli* strain isolated out of calf diarrhea: Characterization and discussion for the need to adapt recent vaccines against neonatal calf diarrhea. Vlaams Diergeneeskd Tijdschr.

[5] Fleckenstein J.M., Hardwidge P.R., Munson G.P., Rasko D.A., Sommerfelt H., Steinsland H. (2010). Molecular mechanisms of enterotoxigenic *Escherichia coli* infection. Microbes Infect..

[6] Nagy B., Fekete P.Z. (1999). Enterotoxigenic *Escherichia coli* (ETEC) in farm animals. Vet. Res..

[7] Sun J., Chen W., Yuan Z. (2022). Characterization of Intestinal Microbiota in Lambs with Different Susceptibility to *Escherichia coli* F17. Vet. Sci..

[8] Ploegh H.L., Orr H.T., Strominger J.L. (1981). Major histocompatibility antigens: The human (HLA-A, -B, -C) and murine (H-2K, H-2D) class I molecules. Cell.

[9] Cole A.M., Liao H.I., Stuchlik O., Tilan J., Pohl J., Ganz T. (2002). Cationic polypeptides are required for antibacterial activity of human airway fluid. J. Immunol..

[10] Chen W., Lv X., Zhang W., Hu T., Cao X., Ren Z., Getachew T., Mwacharo J.M., Haile A., Sun W. (2022). Insights into Long Non-Coding RNA and mRNA Expression in the Jejunum of Lambs Challenged with *Escherichia coli* F17. Front. Vet. Sci..

[11] Xie J., Wang Y., Freeman M.E., Barlogie B., Yi Q. (2003). Beta 2-microglobulin as a negative regulator of the immune system: High concentrations of the protein inhibit in vitro generation of functional dendritic cells. Blood.

[12] Hofbauer D., Mougiakakos D., Broggini L., Zaiss M., Büttner-Herold M., Bach C., Spriewald B., Neumann F., Bisht S., Nolting J. (2021). β2-microglobulin triggers NLRP3 inflammasome activation in tumor-associated macrophages to promote multiple myeloma progression. Immunity.

[13] Ge L., Zou S., Yuan Z., Chen W., Wang S., Cao X., Lv X., Getachew T., Mwacharo J.M., Haile A. (2021). Sheep β-Defensin 2 Regulates *Escherichia coli* F17 Resistance via NF-κB and MAPK Signaling Pathways in Ovine Intestinal Epithelial Cells. Biology.

[14] Livak K.J., Schmittgen T.D. (2001). Analysis of relative gene expression data using real-time quantitative PCR and the 2^−ΔΔCT^ method. Methods.

[15] Canizalez-Roman A., Flores-Villasenor H.M., Gonzalez-Nunez E., Velazquez-Roman J., Vidal J.E., Muro-Amador S., Alapizco-Castro G., Diaz-Quinonez J.A., Leon-Sicairos N. (2016). Surveillance of Diarrheagenic *Escherichia coli* strains isolated from diarrhea cases from children, adults and elderly at Northwest of Mexico. Front. Microbiol..

[16] Le Bouguénec C., Bertin Y. (1999). AFA and F17 adhesins produced by pathogenic *Escherichia coli* strains in domestic animals. Vet. Res..

[17] Ngeleka M., Godson D., Vanier G., Desmarais G., Wojnarowicz C., Sayi S., Huang Y., Movasseghi R., Fairbrother J.M. (2019). Frequency of *Escherichia coli* virotypes in calf diarrhea and intestinal morphologic changes associated with these virotypes or other diarrheagenic pathogens. J. Vet. Diagn. Investg..

[18] Johnson J.R., Russo T.A. (2018). Molecular Epidemiology of Extraintestinal Pathogenic *Escherichia coli*. EcoSal Plus.

[19] Bjorkman P.J., Saper M.A., Samraoui B., Bennett W.S., Strominger J.L., Wiley D.C. (1987). Structure of the human class 1 histocompativility antigen, HLA-A2. Nature.

[20] Pérarnau B., Siegrist C.A., Gillet A., Vincent C., Kimura S., Lemonnier F.A. (1990). β2-microglobulin restriction of antigen presentation. Nature.

[21] Kim J.Y., Park S.C., Lee J.K., Choi S.J., Hahm K.-S., Park Y. (2012). Novel antibacterial activity of beta(2)-microglobulin in human amniotic fluid. PLoS ONE.

[22] Chiou S.J., Ko H.J., Hwang C.C., Hong Y.R. (2021). The Double-Edged Sword of β2-Microglobulin in Antibacterial Properties and Amyloid Fibril-Mediated Cytotoxicity. Int. J. Mol. Sci..

[23] Huang W.-C., Wu D., Xie Z., Zhau H.E., Nomura T., Zayzafoon M., Pohl J., Hsieh C.-L., Weitzmann M.N., Farach-Carson M.C. (2006). β2-microglobulin is a signaling and growth-promoting factor for human prostate cancer bone metastasis. Cancer Res..

[24] Molenaar B., Timmer L.T., Droog M., Perini I., Versteeg D., Kooijman L., Monshouwer-Kloots J., de Ruiter H., Gladka M.M., van Rooij E. (2021). Single-cell transcriptomics following ischemic injury identifies a role for B2M in cardiac repair. Commun. Biol..

[25] Chen W., Lv X., Cao X., Yuan Z., Wang S., Getachew T., Mwacharo J.M., Haile A., Quan K., Li Y. (2023). Integration of the Microbiome, Metabolome and Transcriptome Reveals *Escherichia coli* F17 Susceptibility of Sheep. Animals.

