# Supplementary material for: Beta-2-Microglobulin Regulates Sheep Susceptibility to Escherichia coli F17b in Intestinal Epithelial Cells
Source: Vet Sci. 2026 Mar 9;13(3):252. doi: 10.3390/vetsci13030252 (PMC13030307; doi:10.3390/vetsci13030252)
Supplement: Supplementary file 1 [file vetsci-13-00252-s001.zip › vetsci-4065097-supplementary.pdf]

Table S1. Virulence gene profile of *E. coli F17b* strain DN1401 based on whole-genome sequencing.

Gene Category	Gene	Presence	Notes
ETEC-associated	eltA, eltB (LT)	Absent	Heat-labile enterotoxin genes
	estA (STa), estB (STb)	Absent	Heat-stable enterotoxin genes
	astA (EAST1)	Present	Enteroaggregative heat-stable enterotoxin 1
	paa	Present	Porcine attaching-effacing associated protein
	papC	Present	P fimbrial assembly protein (usher)
NTEC-associated	cnf1, cnf2, cnf3	Absent	Cytotoxic necrotizing factor genes
	cdt-III, cdt-IV	Absent	Cytolethal distending toxin genes
	afa operon	Absent	Afimbrial adhesin operon
Fimbrial genes	F17b-A, F17b-G	Present	F17b fimbrial subunit genes

Data source: Unpublished data from our laboratory.

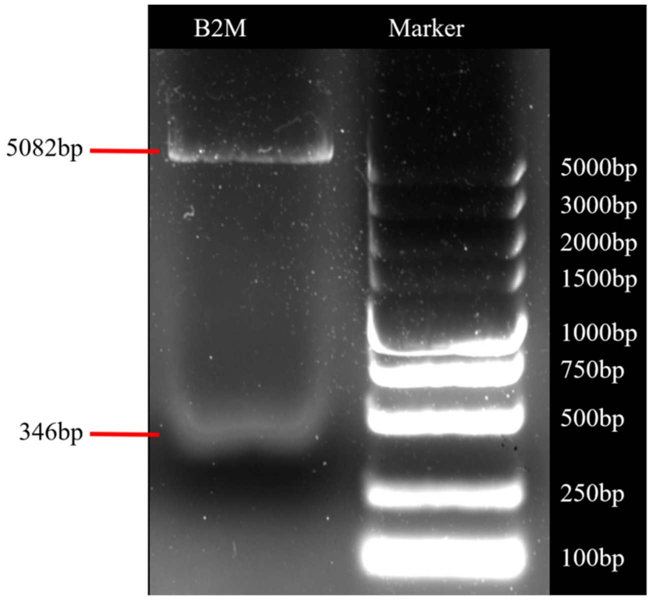

Figure S1. Products of the pcDNA3.1(+)-B2M vector after dual digestion with BamH Ⅰ and HindⅢ.

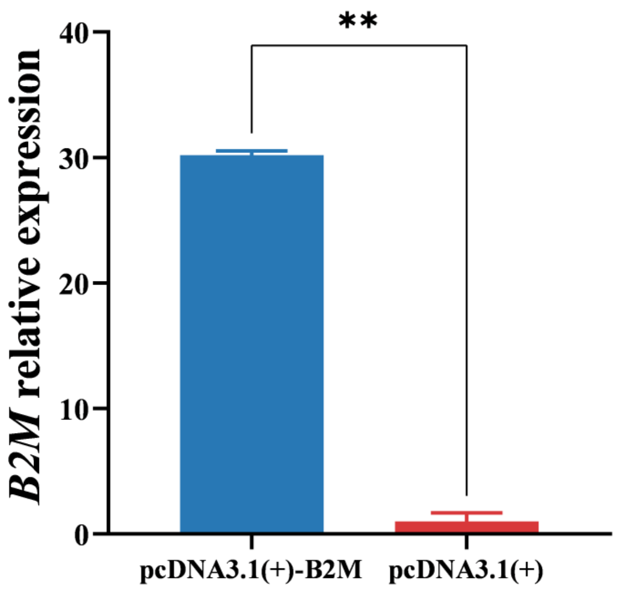

Figure S2. mRNA expression levels of B2M in Hu sheep IECs following overexpression. *P < 0.05, **P < 0.01. Data are presented as the mean ± SD of three independent biological replicates (n = 3).

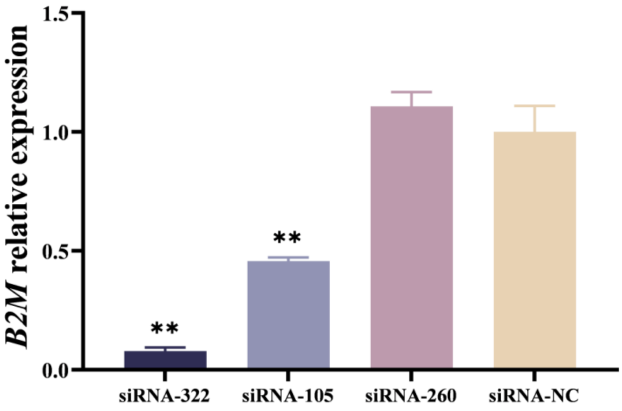

Figure S3. Levels of B2M mRNA expression.. *$P < 0.05$, **$P < 0.01$. Data are presented as the mean ± SD of three independent biological replicates (n = 3).
